# The Neuroprotective Effect of *Hericium erinaceus* Extracts in Mouse Hippocampus after Pilocarpine-Induced Status Epilepticus

**DOI:** 10.3390/ijms20040859

**Published:** 2019-02-16

**Authors:** Hyun-Jong Jang, Ji-Eun Kim, Kyoung Hoon Jeong, Sung Chul Lim, Seong Yun Kim, Kyung-Ok Cho

**Affiliations:** 1 Department of Physiology, Department of Biomedicine and Health Sciences, Catholic Neuroscience Institute, College of Medicine, The Catholic University of Korea, Seoul 06591, Korea; hjjang@catholic.ac.kr; 2 Department of Pharmacology, Department of Biomedicine and Health Sciences, Catholic Neuroscience Institute, College of Medicine, The Catholic University of Korea, Seoul 06591, Korea; jonin12@naver.com (J.-E.K.); jeongkh81@yuhs.ac (K.H.J.); syk@catholic.ac.kr (S.Y.K.); 3 Department of Neurology and Epilepsy Research Institute, Yonsei University College of Medicine, Seoul 03722, Korea; 4 Department of Neurology, St. Vincent’s Hospital, College of Medicine, The Catholic University of Korea, Seoul 06591, Korea; sclim@catholic.ac.kr; 5 Institute of Aging and Metabolic Diseases, The Catholic University of Korea, Seoul 06591, Korea

**Keywords:** *Hericium erinaceus*, status epilepticus, neuroprotection, anti-inflammation, cyclooxygenase 2, pilocarpine

## Abstract

*Hericium erinaceus* (HE), a culinary-medicinal mushroom, has shown therapeutic potential in many brain diseases. However, the role of HE in status epilepticus (SE)-mediated neuronal death and its underlying mechanisms remain unclear. We investigated the neuroprotective effects of HE using a pilocarpine-induced SE model. Male C57BL/6 mice received crude extracts of HE (60 mg/kg, 120 mg/kg, or 300 mg/kg, p.o.) for 21 d from 14 d before SE to 6 d after SE. At 7 d after SE, cresyl violet and immunohistochemistry of neuronal nuclei revealed improved hippocampal neuronal survival in animals treated with 60 mg/kg and 120 mg/kg of HE, whereas those treated with 300 mg/kg of HE showed similar neuronal death to that of vehicle-treated controls. While seizure-induced reactive gliosis, assessed by immunohistochemistry, was not altered by HE, the number of hippocampal cyclooxygenase 2 (COX2)-expressing cells was significantly reduced by 60 and 120 mg/kg of HE. Triple immunohistochemistry demonstrated no overlap of COX2 labeling with Ox42, in addition to a decrease in COX2/GFAP-co-immunoreactivity in the group treated with 60 mg/kg HE, suggesting that the reduction of COX2 by HE promotes neuroprotection after SE. Our findings highlight the potential application of HE for preventing neuronal death after seizures.

## 1. Introduction

Temporal lobe epilepsy (TLE) is one of the most common acquired epilepsies in adults [1]. TLE can be defined as unexpected recurrent seizures that can occasionally result in prolonged seizure activities, status epilepticus (SE). To understand the pathophysiologic mechanisms of human TLE, various animal models, including pilocarpine-induced SE, have been used to replicate neuronal death, reactive gliosis, inflammation, and neurogenesis, which are characteristic features of TLE [2]. Although extensive efforts have been made to determine the molecular mechanisms of TLE, it is still a devastating disease without a cure. Moreover, current dietary therapies that have been proven to be effective for treating epilepsy are high-fat diets such as the ketogenic diet or Atkins diet [3]. Unlike many other diseases where various nutritional supplements and functional foods are widely utilized to improve therapeutic efficacy [4], there are few medicinal foods applicable for epilepsy. Therefore, it is essential to develop both pharmaceutical drugs and functional foods for improved treatment of TLE.

*Hericium erinaceus* (HE), also known as Lion’s Mane or Yamabushitake, is an edible medicinal mushroom that has shown various beneficial effects on a wide range of diseases including cancer, diabetes, dyslipidemia, inflammatory bowel diseases, and infection [5,6,7,8,9,10]. In the central nervous system (CNS), HE could play important roles in alleviating ischemic stroke, Alzheimer’s, and Parkinson’s disease [11,12,13,14,15]. Moreover, we recently reported that chronic HE administration could attenuate anxiety and depressive behaviors in mice [16], which has been further supported by work from other groups [17,18]. HE consists of many bioactive ingredients including erinacines, hericerins, erinaceolactones, glycoproteins, and polysaccharides [19], which have been reported to be associated with increased nerve growth factor (NGF) production [20], enhanced hippocampal neurogenesis [16], and the reduction of endoplasmic reticulum (ER) stress [11,21], oxidative stress [11], excitotoxicity [22,23], and inflammation [9,10]. Since acute seizures induce marked excitotoxicity, oxidative and ER stress, inflammation, and aberrant hippocampal neurogenesis [2,24], HE may be an attractive candidate as a functional food for ameliorating pathophysiologic features of TLE. Therefore, in the present study, we investigated whether HE can have an impact on neuroprotection against pilocarpine-induced SE and its underlying mechanisms, highlighting the potential application of HE administration in TLE.

## 2. Results

### 2.1. HE Administration (60 and 120 Mg/kg) Decreased Hippocampal Cell Death after Pilocarpine-Induced SE

**Figure 1:**
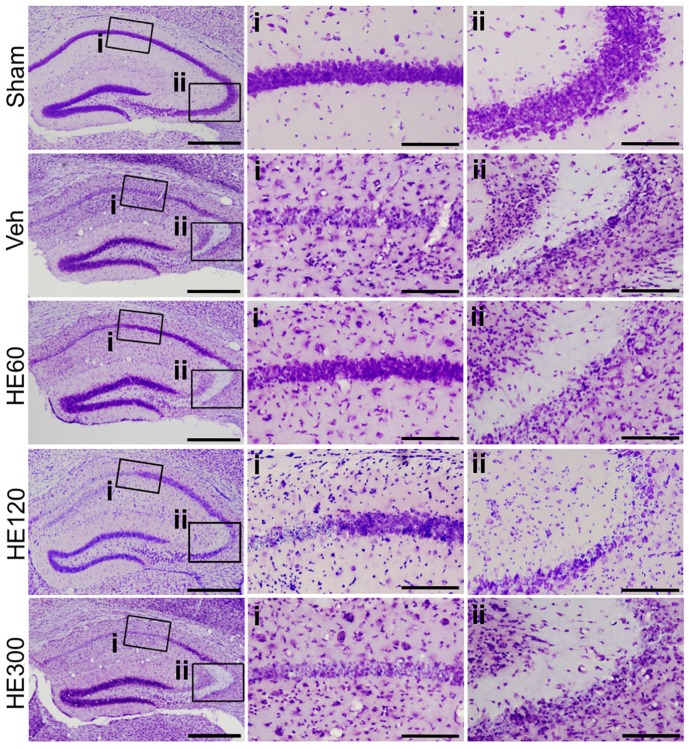
*Hericium erinaceus* (HE) administration at 60 mg/kg and 120 mg/kg decreased hippocampal cell deaths after pilocarpine-induced status epilepticus. Brain sections were stained with cresyl violet. (**i**) Magnified photomicrographs of CA1 subfield of the hippocampus, marked with a rectangle in the left picture. (**ii**) Magnified photomicrographs of CA3 subfield of the hippocampus, marked with a rectangle in the far-left picture. Note that vehicle (Veh)-treated animals showed extensive cell death in the CA1 and CA3 subfields of the hippocampus, compared to sham. HE treatment at 60 and 120 mg/kg could prevent cell death in CA1 but not CA3 subfield of the hippocampus, whereas 300 mg/kg of HE administration showed similar cell death with the group treated with vehicle. Scale bars in the left column: 500 μm, scale bars in the middle column: 100 μm, scale bars in the right column: 100 μm.

Hippocampal cell survival following pilocarpine-induced SE was assessed by cresyl violet staining. Compared to sham, which showed healthy, intact cells, vehicle-treated animals showed a lot of pyknotic cells in the pyramidal cell layer of the CA1 and CA3 subfields of the hippocampus at 7 day after pilocarpine injection (Figure 1). When 60 and 120 mg/kg of HE was administered for 21 day starting from 14 day before pilocarpine injection to 6 day after SE, there were surviving pyramidal neurons in the lateral CA1 subfield of the hippocampus, although cell death was still detected in the CA3 subfield of the hippocampus as well as in the hilar region (Figure 1). However, in the group that received 300 mg/kg HE, cell death was similar to that of vehicle-treated controls, suggesting the dosage of HE is critical for the protective effects against pilocarpine-induced seizures.

### 2.2. 60 and 120 Mg/kg of HE Treatment Showed Significant Hippocampal Neuroprotection after Acute Seizures

**Figure 2:**
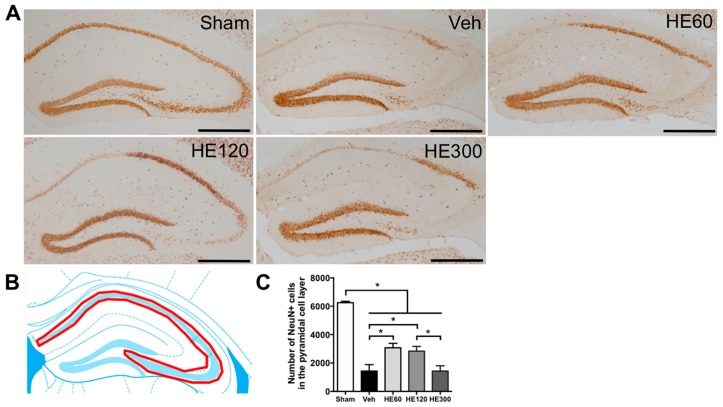
HE treatment at 60 mg/kg resulted in significant hippocampal neuroprotection after pilocarpine-induced status epilepticus. (**A**) Representative photomicrographs showing immunohistochemistry to neuronal nuclei (NeuN) in sham, vehicle (Veh)-, 60 mg/kg of HE- (HE60), 120 mg/kg of HE- (HE120), and 300 mg/kg of HE-treated groups (HE300). Scale bar: 500 μm. (**B**) A drawing of the hippocampus indicating the region for the quantitative analysis of neuronal survival (shown in red). (**C**) A graph showing the number of NeuN-expressing cells in the pyramidal cell layer. Note that Veh-treated group showed a marked reduction in the number of NeuN-positive neurons compared to sham at 7 d after status epilepticus (SE). However, 60 mg/kg and 120 mg/kg of HE administration significantly prevented neuronal death against SE, whereas 300 mg/kg of HE showed the similar number of surviving pyramidal neurons compared to Veh-treated animals. Data are shown as mean ± SEM. *n* = 6 (sham), *n* = 9 (Veh), *n* = 6 (HE60), *n* = 9 (HE120), *n* = 6 (HE300). * *p* < 0.05; one-way ANOVA with Tukey’s post-hoc test.

For accurate quantitative analysis of the neuroprotective effects of HE administration against SE, we stained hippocampal tissue sections with the neuronal marker, neuron-specific nuclear protein (NeuN). Consistent with cresyl violet results, NeuN-positive cells in the CA1 and CA3 pyramidal cell layer were not detected after pilocarpine-induced SE (Figure 2A). However, 60 mg/kg and 120 mg/kg of HE administration could save lots of pyramidal neurons in the lateral CA1 subfield of the hippocampus at 7 day after acute seizures, whereas in the group that received 300 mg/kg of HE, NeuN-expressing cells were only observed in the CA2 subfield of the hippocampus with a few CA1 and CA3 neurons. When we counted the number of NeuN-immunoreactive cells in the pyramidal cell layer (Figure 2B), we found that pilocarpine-induced SE resulted in marked reduction in the number of pyramidal neurons (Figure 2C). However, 60 and 120 mg/kg of HE treatment significantly increased the number of NeuN-expressing cells in the pyramidal cell layer, indicating the neuroprotective effects of HE after acute seizures.

### 2.3. HE Administration Did Not Affect Reactive Gliosis after Acute Seizures

**Figure 3:**
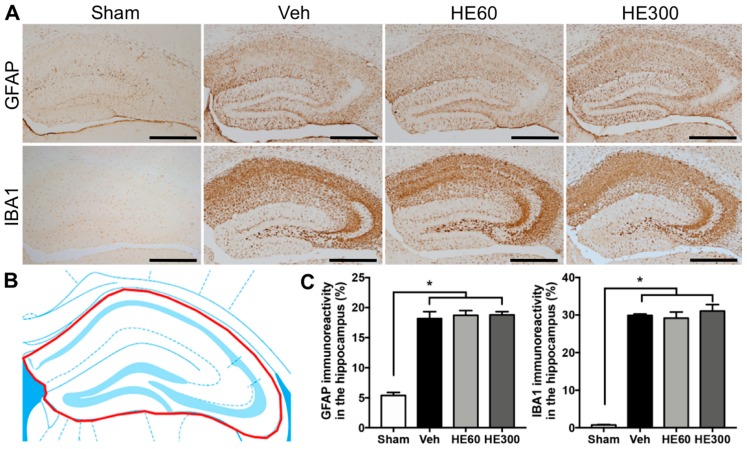
Reactive gliosis after pilocarpine-induced SE was not altered by either low- or high-dose HE administration. (**A**) Representative photomicrographs showing immunohistochemistry to glial fibrillary acidic protein (GFAP) and ionized calcium-binding adapter molecule 1 (IBA1) in sham, vehicle (Veh)-, 60 mg/kg HE- (HE60), and 300 mg/kg HE-treated groups (HE300). Scale bar: 500 μm. (**B**) A drawing of the hippocampus indicating the region for the quantitative analysis of reactive gliosis (shown in red). (**C**) Graphs showing the immunoreactivity to GFAP and IBA1 in the hippocampus. Note that both GFAP and IBA1 immunoreactivity were significantly increased by SE, but there were no differences in the glial activation among Veh-, HE60-, and HE300-treated groups. Data are shown as mean ± SEM. *n* = 6 (sham), *n* = 10 (Veh), *n* = 8 (HE60), *n* = 6 (HE300). * *p* < 0.05; one-way ANOVA with Tukey’s post-hoc test.

As a next step, we investigated the effects of oral HE administration on reactive gliosis after pilocarpine-induced SE. Immunohistochemistry with glial fibrillary acidic protein (GFAP) or ionized calcium-binding adapter molecule 1 (IBA1), a marker for astrocytes and microglia, respectively, showed that acute seizure activities remarkably increased the immunoreactive areas expressing GFAP and IBA1 in the hippocampus (Figure 3A). When we quantitatively analyzed glial activation in the hippocampus after SE (Figure 3B), there were no significant changes in reactive astrocytosis and microglial activations by HE treatment (Figure 3C), suggesting that HE administration did not affect the glial activation induced by acute seizures.

### 2.4. HE Administration (60 and 120 Mg/kg) Reduced the Number of Cyclooxygenase 2 (COX2)-Expressing Cells in the Hippocampus after Acute Seizures

**Figure 4:**
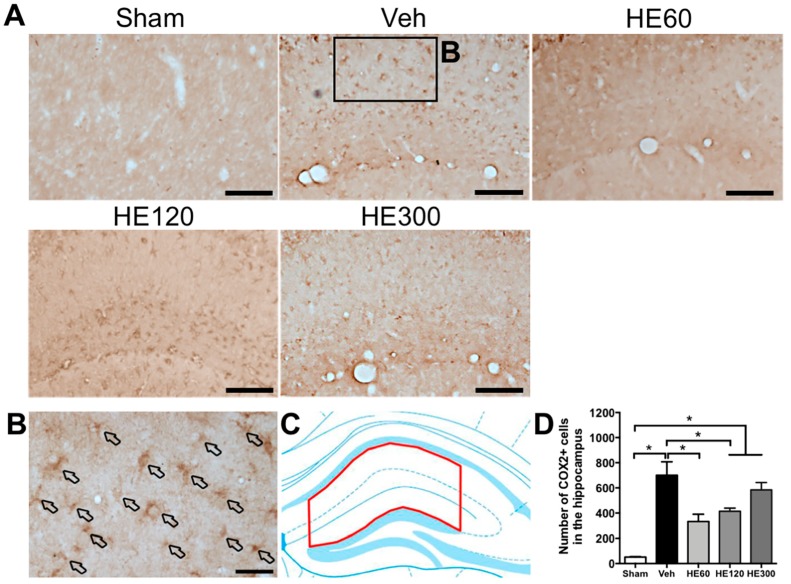
HE administration at 60 and 120 mg/kg reduced the number of cyclooxygenase 2 (COX2)-expressing cells in the hippocampus after pilocarpine-induced SE. (**A**) Representative photomicrographs showing immunohistochemistry to COX2 in sham, vehicle (Veh)-, 60 mg/kg HE- (HE60), 120 mg/kg HE- (HE120), and 300 mg/kg HE-treated groups (HE300). Scale bar: 100 μm. (**B**) A magnified photomicrograph showing COX2-expressing cells in the region marked as a rectangle in Veh-treated group. Arrows indicate various COX2-expressing cells in the hippocampus. Scale bar: 20 μm. (**C**) A drawing of the hippocampus indicating the region for the quantitative analysis of COX2 expression (shown in red). (**D**) A graph showing the number of COX2-expressing cells in the hippocampus. Note that SE induced marked upregulation of COX2-expressing cells shown in Veh-treated group. HE administration at 60 and 120 mg/kg led to significantly fewer COX2-positive cells in the hippocampus than the Veh-treated group, whereas 300 mg/kg HE treatment resulted in similar COX2 activation compared to the Veh-treated group. Data are shown as mean ± SEM. *n* = 7 (sham), *n* = 9 (Veh), *n* = 8 (HE60), *n* = 7 (HE120), *n* = 6 (HE300). * *p* < 0.05; one-way ANOVA with Tukey’s post-hoc test.

We then sought to identify target molecules of which expression was correlated with NeuN-staining results. Immunohistochemistry to cyclooxygenase 2 (COX2) revealed that vehicle-treated group showed many COX2-expressing cells in the hippocampus after SE, while there were fewer COX2-positive cells in the group treated with 60 mg/kg and 120 mg/kg of HE (Figure 4A). Most of the COX2-expressing cells induced by acute seizures were scattered in the stratum radiatum and lacunosum-moleculare of the CA1 subfield, which have multiple processes (Figure 4B). When we quantitatively analyzed COX2-expressing cells after SE (Figure 4C), the number of COX2-immunoreactive cells showed significantly increased levels in the vehicle-treated group, which were markedly reduced by treatment with 60 and 120 mg/kg of HE (Figure 4D). However, COX2 immunoreactivity was upregulated in the group treated with 300 mg/kg of HE compared to sham, consistent with our findings showing neuronal death (Figure 4D).

### 2.5. Low-dose HE Treatment Suppressed Hippocampal COX2-Expressing Glial Cells after SE

**Figure 5:**
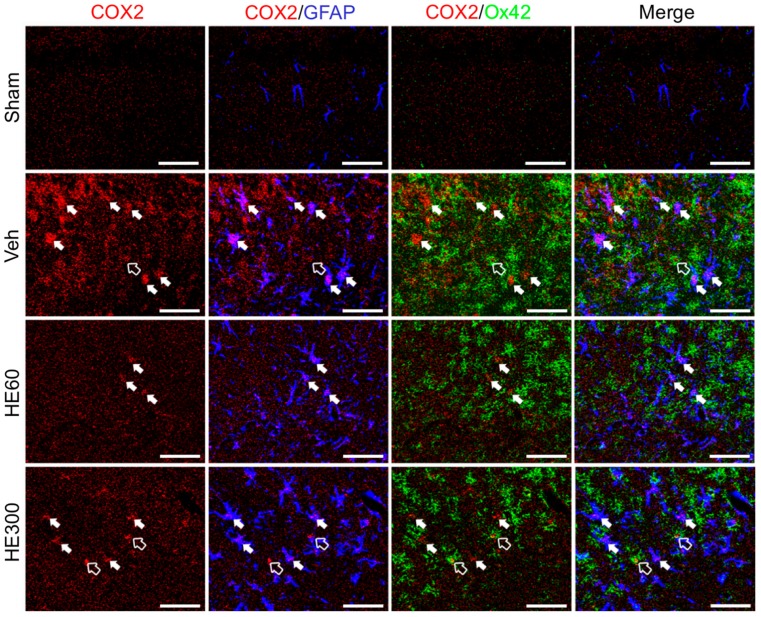
HE treatment at 60 mg/kg suppressed hippocampal COX2-expressing glial cells after pilocarpine-induced SE. Triple immunohistochemistry for COX2 (red), GFAP (blue), and Ox42 (green) demonstrated that COX2-expressing cells in the Veh-treated group turned out to be GFAP-positive astrocytes (filled arrows) and Ox42-positive microglia (hollow arrow). In the group treated with 60 mg/kg HE, COX2/Ox42-coexpressing cells were not observed, and COX2/GFAP immunoreactivity was reduced. However, in the group treated with 300 mg/kg HE, COX2-expressing astrocytes and microglia were comparable with those in the Veh-treated group. Scale bar: 50 μm.

Since the morphology of COX2-expressing cells was similar to that of glial cells, triple immunofluorescence with COX2, GFAP, and Ox42, another microglial marker, was performed to identify the phenotype of COX2-expressing cells (Figure 5). Compared to sham-controls where no COX2/GFAP or COX2/Ox42-coexpressing cells were observed, many COX2 signals in the CA1 subfield of the hippocampus were co-labeled with GFAP in the vehicle-treated group, in addition to the colocalization of COX2 and Ox42 to a lesser degree. Interestingly, when 60 mg/kg of HE was administered, COX2 labeling did not overlap with Ox42, which was still detected in the group treated with 300 mg/kg of HE, suggesting that microglial COX2 expression can be critical for the neuroprotective effects of low-dose HE administration. Moreover, COX2/GFAP-coexpressing cells that were markedly increased by acute seizures had a decreasing tendency upon 60 mg/kg of HE administration, which was sustained in the group treated with 300 mg/kg of HE. Taken together, these data demonstrated that low-dose administration of HE reduced COX2 expression in glial cells after pilocarpine-induced SE.

## 3. Discussion

Current treatment options available for patients with TLE are medication with anti-epileptic drugs, neuro-stimulation, and surgical resection of epileptic foci [25,26,27,28]. Two-thirds of patients can successfully control recurrent seizures by taking anti-epileptic drugs, while deep brain stimulation, vagal nerve stimulation or surgery can be considered for the remaining one-third suffering from intractable epilepsy [28]. Moreover, extensive neuronal damage, inflammation, and aberrant neurogenesis observed in the patients with epilepsy are considered to contribute to the generation of chronic seizures, urging new drug developments alleviating pathologic manifestations in a comprehensive manner [29,30,31]. However, due to the serious adverse effects of current drug therapies and the limitations of alternative treatment methods, screening of various nutritional supplements and functional foods is required for potential supplementation in TLE. Currently, several natural products, including curcumin, resveratrol, berberine, baicalein, and omega-3 fatty acids have demonstrated various beneficial effects in animal models of epilepsy [32,33,34,35,36]. In line with these reports, we showed that HE supplementation could promote neuroprotection against SE, providing a new nutraceutical candidate for TLE. Previous reports using in vitro glutamate-induced excitotoxic injury also demonstrated that HE treatment could improve the viability of PC12 neuronal cells [22,23], supporting our findings. Given that the clinically available dosage for HE can be approximately 40 mg/kg if the height of the study participants was presumed to be 160 cm [17], the observed neuroprotective effects from oral administration of 60 mg/kg HE in our study can be useful in terms of the potential clinical application of HE in epilepsy.

Interestingly, when we examined the dose-dependence of HE in neuroprotective effects against SE, we found that 60 and 120 mg/kg of HE significantly reduced pilocarpine-induced neuronal death in the hippocampus, whereas 300 mg/kg HE resulted in a similar level of cellular damage compared to that from vehicle-treated animals. These data suggest that there is an effective dose window for HE to facilitate neuronal survival against prolonged seizure activities. To understand the underlying mechanisms, we assessed a pro-inflammatory factor, COX2, in the hippocampus after SE. In agreement with our cell death data, the number of COX2-expressing glial cells was significantly decreased by 60 and 120 mg/kg of HE administration, while it was maintained at high levels in the group treated with 300 mg/kg of HE. Supporting our findings, anti-inflammatory effects of HE have been reported in many diseases, including stroke, depression, inflammatory bowel disease, and ulcerative colitis [9,10,12,18]. Specifically, HE and its bioactive component, erinacine A, could inhibit the expression of inflammatory cytokines, such as interleukin (IL)-1β, IL-6, or tumor necrosis factor (TNF)-α [9,10,12]. Moreover, the expression of nuclear factor-kappa B (NF-κB) was also normalized by HE treatment [9,18], in addition to the downregulation of COX2 expression in macrophages [37]. As the COX2 enzyme is rapidly induced after acute seizures and contributes to neuronal death in epilepsy [38,39,40], the suppression of COX2 expression by 60 and 120 mg/kg of HE treatment can promote neuroprotection against pilocarpine-induced SE. One interesting observation we made was that low-dose HE could reduce glial COX2 expression without affecting seizure-induced gliosis. We speculate that HE treatment may alter the glial characteristics towards being less inflammatory, even though it cannot manipulate reactive gliosis after acute seizures. However, further studies are required to elucidate the molecular mechanisms of COX2 activation and glial behaviors that are differentially regulated by HE dosage regimens.

HE contains many biologically active ingredients including hericerins, erinacines, erinaceolactones, glycoproteins, and polysaccharides, in addition to new compounds still being identified [19,41]. Although the bioactive compounds responsible for the pro-survival effects of HE are not fully understood, several studies have sought to identify functional molecules in HE [12,21,22,42,43,44]. For example, erinacine A, one of the major diterpenoids, could inhibit glutamate-induced apoptosis and ischemic neuronal deaths [12,22]. Enhanced NGF production and the suppression of inflammatory mediators such as inducible nitric oxide synthase, IL-1β, and TNF-α by erinacine A are supposed to mediate neuroprotective effects of HE [12,42]. Moreover, erinacine A can suppress oxidative stress by reducing nitrotyrosine and the expression of CCAAT enhancer-binding protein homologous protein (CHOP) [12], in addition to the activation of TrkA and Erk1/2 pathways [41], implying that erinacine A in our crude HE extracts may exert neuroprotection against SE. However, as linoleic acids isolated from HE could also promote neuronal survival [21], it is plausible that multiple functional compounds can generate synergistic effects, providing neuroprotection in epilepsy. Since polysaccharides and glycoproteins from HE, on the other hand, could increase cancer cell death [43,44], HE-induced neuroprotection in our study may be the net outcome of various compounds leading to promotion or inhibition of cell survival. Therefore, it will be interesting to investigate the impact of each bioactive ingredient of HE on seizure-induced neuronal death, in addition to the discovery of new compounds improving neuronal survival in future studies.

To enhance neuroprotective potentials of HE after acute seizures, utilizing nanodelivery systems can be considered, especially when the essential bioactive component of HE is established. There are two types of lipid nanoparticles called solid lipid nanoparticles and nanostructured lipid carriers, depending on the function of the lipids forming nanoparticle matrix [45]. By mixing lyotropic lipid, monoolein, with assorted emulsifiers and water, various types of liquid crystalline nanocarriers can be generated [46,47,48,49,50]. Different materials can also be used to encapsulate target compounds in lipid vehicles for improving delivery efficiencies or the drug release in a controlled fashion [51,52,53]. As lipid dispersions can dissolve highly hydrophobic natural compounds and often stabilize chemical instability, they can improve the bioavailability of the natural compounds, maintaining therapeutic concentrations for a longer duration [49,54]. These effects can help to reduce side effects and the dosing frequency of the natural compounds, which will greatly accelerate the development of health supplements for epilepsy.

In summary, we showed in the present study that 60 and 120 mg/kg of HE administration could attenuate hippocampal neuronal death 7 day after pilocarpine-induced SE. However, neuroprotective effects were not observed with the high-dose administration of HE, suggesting that there are optimal dose ranges of HE for neuroprotection against seizure activities. Although we did not perform thorough evaluations on the acute seizure severity [55], it is noteworthy that the progression of acute seizure activity was comparable among four different groups based on the behavioral assessment (data not shown), which gave us a rationale to examine neuroprotective effects of HE. While reactive astrocytosis and microglial activation induced by SE were not altered by HE treatment, the number of COX2-expressing cells was significantly decreased by 60 and 120 mg/kg of HE administration, whereas 300 mg/kg of HE showed comparable COX2 upregulation to that of the vehicle-treated group. Finally, we demonstrated that COX2-expressing microglia were not present upon administration of 60 mg/kg of HE, in addition to a reduction of COX2-expressing astrocytes, suggesting that neuroprotective effects of low-dose HE supplementation can be associated with COX2 expression.

## 4. Materials and Methods

### 4.1. Pilocarpine-Induced SE

Male C57BL/6 mice (6 weeks old, Koatech, Kyunggi-do, Korea) were housed at a standard temperature (22 ± 1 °C) and in a light-controlled environment (lights on from 8:00 am to 8:00 pm) with ad libitum access to food and water. Animal experiments were approved by the Institutional Animal Care and Use Committee (IACUC) in School of Medicine, The Catholic University of Korea (CUMS-2017-0116-03) and were carried out in accordance with the National Institutes of Health Guideline for the Care and Use of Laboratory Animals (NIH Publication No. 80–23, revised 1996). Animals were treated as previously described [56]. Briefly, mice were given atropine methyl nitrate (2 mg/kg, i.p., Tokyo Chemical Industry Co., Tokyo, Japan) and terbutaline hemisulfate (2 mg/kg, i.p., Sigma–Aldrich, St. Louis, MO, USA) 30 min before the injection of pilocarpine hydrochloride (280 mg/kg, i.p., Sigma–Aldrich). After pilocarpine administration, mice were closely monitored for approximately 6 h to evaluate the severity and length of behavioral seizures. The seizure stage was determined using the Racine scale [57]: stage 1, facial clonus; stage 2, head nodding; stage 3, forelimb clonus; stage 4, rearing; and stage 5, rearing and falling. Only animals that had stage 5 generalized tonic–clonic seizures (rearing and falling) were considered to show SE and were selected for the study. After 2 h of SE, diazepam (10 mg/kg, i.p.) was administrated to quell seizures. Sham-manipulated animals were injected with the same dose of atropine and terbutaline as above but were then injected with saline instead of pilocarpine. To facilitate recovery, all experimental animals were housed in an incubator (30 ± 1 °C) and were given water-moistened chow for 5 day until they gained weight. The animals were then returned to standard cages and sacrificed at 7 day after SE onset (*n* = 6–10 for each group).

### 4.2. Administration of HE

Crude extracts of HE were kindly provided by Young-Ock Kim at Rural Development Administration in South Korea [37]. Briefly, HE was extracted with 70% ethanol for 3 d at room temperature, followed by evaporation in vacuo to generate the crude extract of HE (yield: 32.6%). HE (60, 120, or 300 mg/kg) or saline was freshly prepared every day immediately before the daily oral administration by gavage. HE treatment was maintained for 21 day from 14 day before pilocarpine injection until 6 day after SE.

### 4.3. Staining

Mice were deeply anesthetized with chloral hydrate (500 mg/kg, i.p.) and transcardially perfused with saline followed by 4% paraformaldehyde in 0.1 M sodium phosphate buffer (PB, pH 7.4, Sigma–Aldrich). Brains were removed and post-fixed in paraformaldehyde for 24 h and then dehydrated with 30% sucrose in 0.1 M PB for 3 day. Brains were embedded in Tissue-Tek (Sakura Finetechnical, Tokyo, Japan) for cryoprotection and were rapidly frozen with liquid nitrogen. Using a cryostat, 20-μm-thick serial coronal sections were collected at every 6th section at an interval of 120 μm (total 720 μm, between −1.70 and −2.42 from bregma) and were further processed for staining.

Neuronal cell death was evaluated using cresyl violet staining. Briefly, tissue slides were serially hydrated from 100% ethanol (Merck Millipore, Darmstadt, Germany) to tap water. They were then incubated in 0.1% cresyl violet solution (Sigma–Aldrich) for 15 min. After destaining with 95% ethanol containing 0.1% glacial acetic acid (Sigma–Aldrich), the sections were dehydrated in a graded ethanol series (70%–100%), followed by 100% xylene (Sigma–Aldrich), and were mounted with Canada balsam (Sigma–Aldrich). Finally, the sections were observed under light microscopy (BX51, Olympus, Tokyo, Japan).

For immunohistochemistry, floating sections were washed three times (5 min each) in 0.01 M phosphate-buffered saline (PBS, pH 7.4, Sigma–Aldrich). The tissues were then incubated with 3% H_2_O_2_ (Merck Millipore) and 10% methanol (Merck Millipore) in 0.01 M PBS to inhibit endogenous peroxidase activity. Next, the sections were blocked with 10% normal goat serum (Vector Laboratories, Burlingame, CA, USA) in 0.01 M PBS for 1 h and were incubated overnight at 4 °C with an antibody to NeuN (1:100, EMD Millipore, Billerica, MA, USA), GFAP (1:400, EMD Millipore, USA), IBA1 (1:500, Wako Pure Chemicals Inc., Osaka, Japan), or COX2 (1:200, Cayman Chemical Co., Ann Arbor, MI, USA). The next day, the sections were incubated with anti-mouse IgG (1:500, Vector Laboratories) or biotinylated anti-rabbit IgG (1:200, Vector Laboratories) for 2 h at room temperature and were put in an avidin-biotin peroxidase complex solution (1:50, Vector Laboratories) for 1 h. Finally, the sections were visualized with 0.1% diaminobenzidine tetrahydrochloride and 0.005% H_2_O_2_ in 0.05 M Tris–HCl (pH 7.4, Sigma-Aldrich) and were observed using a light microscope.

For triple immunofluorescence, the sections were first incubated overnight at 4 °C with rabbit anti-COX2. The following day, the sections were incubated with Cy3 conjugated anti-rabbit IgG (1:500, Jackson ImmunoResearch, West Grove, PA, USA) for 2 h at room temperature. After being rinsed with 0.1 M PB for 15 min, the sections were incubated overnight at 4 °C with rat anti-Ox42 (1:100, Serotec, Oxford, UK). On the third day, sections were labeled with Alexa fluor 488-conjugated anti-rat (1:300, Invitrogen, Carlsbad, CA, USA) for 2 h and were successively incubated overnight at 4 °C with mouse anti-GFAP (1:400, EMD Millipore). On the fourth day, the sections were labeled with Cy5-conjucated anti-mouse IgG (1:500, Jackson ImmunoResearch) for 2 h at room temperature. The sections were then mounted and observed using a confocal microscope (LSM 510 Meta, Carl Zeiss Co., Ltd., Jena, Germany).

### 4.4. Image Analysis

All quantitative analyses were performed in a double-blind manner. The number of NeuN- or COX2-positive cells was counted in the hippocampal region indicated in Figure 2 and Figure 4, respectively. Then, the results from 6 sections were all added and multiplied by 6 to estimate the total number of immunoreactive cells in the middle one-third of the hippocampus. For GFAP and IBA1 analysis, the percentage of the area of stained cells was compared to the entire hippocampus using Image-Pro Plus software (version 5.1; MediaCybernetics, Silver Spring, MD, USA). Briefly, the background optical density was systematically subtracted from the optical density of each section. The area covered by immunopositive cells was computed as a ratio of the total area delineated.

### 4.5. Statistical Analysis

Data are expressed as mean ± SEM and statistical significance was assessed using GraphPad Prism 7 software (GraphPad Software Inc., La Jolla, CA, USA). For the analysis of NeuN-, GFAP-, and COX2-immunoreactive cells, one-way analysis of variance (ANOVA) and Tukey’s post-hoc test were performed. Moreover, IBA1-expressing cells were analyzed by the Kruskal–Wallis nonparametric test followed by Dunn’s multiple comparisons test, because Gaussian distribution was not assumed. *p* < 0.05 was considered significant.

## Abbreviations

CNS	Central nervous system
COX2	Cyclooxygenase 2
GFAP	Glial fibrillary acidic protein
HE	*Hericium erinaceus*
IBA1	Ionized calcium-binding adapter molecule 1
IL	Interleukin
NeuN	Neuron-specific nuclear protein
NF-κB	Nuclear factor-kappa B
NGF	Nerve growth factor
PB	Phosphate buffer
PBS	Phosphate-buffered saline
SE	Status epilepticus
TLE	Temporal lobe epilepsy
TNF	Tumor necrosis factor
